# Data sharing statements: impact of journal policies across clinical research disciplines

**DOI:** 10.1093/eurheartj/ehaf359

**Published:** 2025-05-30

**Authors:** Daniel Archer, Noah Barks, Mahad Chaudhry, Brody Dennis, Jacob Duncan, Annes Elfar, Taylor Gardner, Eli Paul, Micah Kee, Alicia Ito Ford, Matt Vassar

**Affiliations:** Office of Medical Student Research, Oklahoma State University Center for Health Sciences, 1111 W 17th St., Tulsa, OK 74107, USA; Office of Medical Student Research, Oklahoma State University Center for Health Sciences, 1111 W 17th St., Tulsa, OK 74107, USA; Office of Medical Student Research, Oklahoma State University Center for Health Sciences, 1111 W 17th St., Tulsa, OK 74107, USA; Office of Medical Student Research, Oklahoma State University Center for Health Sciences, 1111 W 17th St., Tulsa, OK 74107, USA; Office of Medical Student Research, Oklahoma State University Center for Health Sciences, 1111 W 17th St., Tulsa, OK 74107, USA; Office of Medical Student Research, Oklahoma State University Center for Health Sciences, 1111 W 17th St., Tulsa, OK 74107, USA; Office of Medical Student Research, Oklahoma State University Center for Health Sciences, 1111 W 17th St., Tulsa, OK 74107, USA; Office of Medical Student Research, Oklahoma State University Center for Health Sciences, 1111 W 17th St., Tulsa, OK 74107, USA; Department of Internal Medicine, Oklahoma State University Medical Center, Tulsa, OK 74127, USA; Office of Medical Student Research, Oklahoma State University Center for Health Sciences, 1111 W 17th St., Tulsa, OK 74107, USA; Department of Psychiatry and Behavioral Sciences, Oklahoma State University Center for Health Sciences, Tulsa, OK 74107, USA; Office of Medical Student Research, Oklahoma State University Center for Health Sciences, 1111 W 17th St., Tulsa, OK 74107, USA; Department of Psychiatry and Behavioral Sciences, Oklahoma State University Center for Health Sciences, Tulsa, OK 74107, USA

**Keywords:** Cross-sectional, Data, Cardiology, Sharing, Reuse, Transparency

## Abstract

**Background and Aims:**

Cardiovascular disease is a leading cause of mortality, with significant investments in research to improve treatment and prevention. Data sharing enhances transparency, reproducibility, and collaboration, yet data sharing statement (DSS) inclusion remains inconsistent. This study evaluates DSS prevalence, content, and influencing factors in high-impact cardiology journals, examines journal policy influence, and assesses data sharing feasibility by contacting authors who indicated data availability.

**Methods:**

A cross-sectional analysis was conducted to assess DSS inclusion in top cardiology, selected general medicine, emergency medicine, and orthopaedic surgery journals. A systematic PubMed search identified clinical studies published from 2020 to 2023. Logistic regression models assessed factors associated with DSS inclusion, while thematic analysis categorized DSS content. Corresponding authors who indicated data availability upon request were contacted to evaluate follow-through.

**Results:**

Among 2941 articles, 1004 (34.14%) included a DSS. Data sharing statement prevalence varied by discipline: cardiology (52%), general medicine (96%), emergency medicine (12%), and orthopedic surgery (14%). Policy enforcement drove DSS inclusion, with post-policy articles significantly more likely to contain a DSS. Funding status, study design, article access, and impact factor also influenced DSS presence. Thematic analysis identified conditional availability and gatekeeping as dominant DSS themes. Of authors who stated data were available upon request, only 31% ultimately provided access.

**Conclusions:**

Data sharing statement inclusion in cardiology research remains inconsistent, with journal policies playing a key role in increasing prevalence. However, real-world data-sharing practices often fall short of stated commitments. Addressing logistical and financial barriers will be essential to improving data availability in cardiology research.

## Structured Graphical Abstract

**Structured Graphical Abstract:**
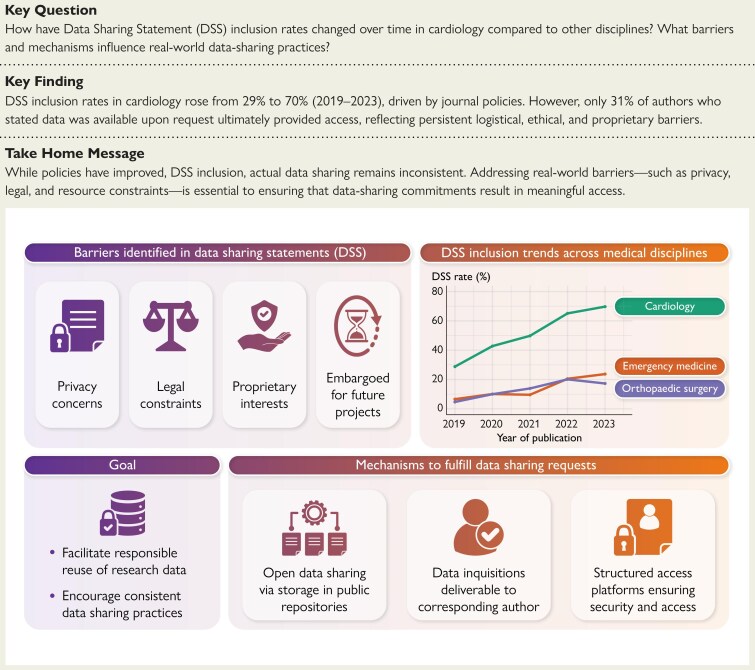

**See the editorial comment for this article ‘Advancing the conversation of data sharing in cardiovascular clinical research’, by A.S. Bhatt and S.D. Solomon, https://doi.org/10.1093/eurheartj/ehaf730.**

## Introduction

Cardiovascular disease (CVD) remains the leading cause of global mortality, responsible for over 30% of deaths worldwide and approximately 1.7 million deaths in Europe in 2021.^1–5^ Additionally, the American Heart Association reports spending on CVD has increased by over $100 billion since 1996 and now stands at $320 billion annually in the US, with spending in Europe surpassing €282 billion in 2021.^5,6^ Given the financial and health implications associated with CVD, the need for high-quality research is essential to developing novel treatment plans in cardiology.^7^ One way to enhance the impact of research is through data sharing, which plays a key role in fostering collaboration, enabling further scientific discovery, and supporting the broader research community.^8–10^

Data sharing involves making data from original research available. The *New England Journal of Medicine* outlines several advantages associated with sharing patient-level data from clinical trials, including the potential for discoveries from secondary analysis, confirmation of results, and the opportunity to conduct meta-analyses from aggregated data sets.^11^ However, data sharing is not a simple task, as demonstrated by a 2024 study that examined specific difficulties with sharing data in cardiology. This study cited barriers such as limited awareness, lack of funding, and time constraints.^12^Furthermore, Assante *et al*.^13^ found hosting costs to be a concern for data storage in public repositories. In contrast, another study found that over two-thirds of researchers would be more than willing to share data if funding agencies assisted with repository expenses.^14^ Ongoing obstacles such as ethical and compliance confusion, the absence of participant consent, and international privacy law discrepancies continue to hinder the practice of data sharing.^12–14^ Understanding how authors, journals, and stakeholders use data sharing statements (DSSs) is a crucial foundation for future research on overcoming data sharing barriers.

Data sharing is often facilitated through DSS or data repositories.^15^ These DSS highlight the availability and accessibility of data used in a study, allowing readers to understand if data is available and what steps or processes are necessary to obtain the data.^16,17^ Even as data sharing policies have become more common in academic publishing, studies suggest that a substantial proportion of manuscripts do not contain DSS, limiting insights into how often data are made available, under what conditions data is made available, or understanding why data may not be available.^9,18,19^

For these reasons, our study aimeds to assess the prevalence of DSS in clinical cardiology studies. We aim to evaluate DSS inclusion, understand how policies affect DSS presence, assess factors associated with DSS inclusion, and identify themes present within cardiology DSS. Additionally, we will contact corresponding authors who state their data is available upon request to understand the feasibility of providing data. By describing the data sharing landscape within cardiology, we aim to advance collaboration and promote open science practices.

## Methods

### Study design, ethics, reporting and reproducibility

We applied systematic review techniques to locate, select, and extract data from primary studies, followed by performing a cross-sectional analysis of the gathered data. The Preferred Reporting Items for Systematic Reviews and Meta-Analyses (PRISMA) guidelines were deemed most appropriate for this research, as opposed to the Strengthening the Reporting of Observational Studies in Epidemiology.^20,21^ The study received approval from the Oklahoma State University Center for Health Sciences Institutional Review Board, with study number 2024110. Additionally, we have made our protocol (including all its versions), analysis scripts, research data, and other study materials available on the Open Science Framework (OSF).^22^

### Search strategy

We selected four top cardiology journals based on impact factor ranking in 2023 from Clarivate’s Journal Citation Reports^TM^ on 5 June 2024. We excluded journals whose aims and scopes did not align with the traditional focus of clinical cardiology. Additionally, each journal needed to have published at least 30 clinical studies to be included. Journals with a cardiology subspecialty focus were not included. Further, we included cardiology-specific publications in the Journal of the American Medical Association (JAMA) Network, NEJM, and The Lancet to provide insight into DSS practices among some of the top general medicine journals. We then conducted a comprehensive literature search in MEDLINE (PubMed), tailored to include only clinical studies. Additional search limits were applied to narrow the publication date range from 1 January 2020, to 31 December 2023, to ensure the data reflects the most recent advancements in cardiology research. Lastly, data from published studies in emergency medicine and orthopaedic surgery were incorporated for analysis—allowing for direct comparison between cardiology, emergency medicine, orthopaedic surgery, and general medical journals.^23,24^ Studies from emergency medicine that were e-published before 2019 were excluded from this study to match the date range of those from cardiology.

### Training

All authors underwent training on study objectives, methodology, and use of the Rayyan systematic review platform (https://www.rayyan.ai/). Prior to full data extraction, two reviewers (D.A., N.B.) independently extracted data from 10 sample articles in a calibration exercise. Their results were reviewed to ensure consistency before proceeding.

### Eligibility criteria

Eligible studies were original clinical research on cardiology procedures or outcomes, including clinical trials, cohort, case control, cross-sectional studies, and case series with novel primary data. We excluded reviews, single-patient case reports, systematic reviews, editorials, commentaries, errata, and studies unrelated to cardiology or lacking new data. Two reviewers (D.A., N.B.) independently screened articles, resolving disagreements with a third (M.C.). While we limited the search to 2020–23, PubMed indexing by e-publication date led to the inclusion of some 2019 studies.

### Primary data extraction

Two authors (D.A., N.B.) independently used a pilot-tested Google Form to extract the following data elements: bibliographic details (PMID, name of journal, article e-publication date), type of study design (see Screening/Eligibility criteria), study topic (diagnostic/screening or treatment/intervention), accessibility and open access status of the journal article, funding source categories (e.g. private, industry, hospital, government, not listed, university, not funded), presence and verbatim extract of the DSS, journal DSS policies, repository details (repository/database name, website URL, data DOI), corresponding author email, and accessibility status of the data (open access, restricted, unavailable, or embargoed). The results were then reconciled via discussion and collaborative review of the extraction source.

### Journal and publisher data sharing statement policy grading

To assess the influence of journal and publisher policies on DSS, we reviewed instructions for authors, submission guidelines, and other webpages on their respective websites that housed data sharing policies. Policies were categorized as ‘Required’, ‘Required for Clinical Trials’, or ‘Recommended’. If no policy was found, it was classified as ‘No Policy’. Verbiage such as ‘required’, ‘must’, ‘need’, ‘mandatory’, and ‘studies will not be considered for publication unless…’ was interpreted as ‘Required’. Terms like ‘encouraged’, ‘should’, and ‘preferred’ were classified as ‘Recommended’. These classifications are based on previous literature.^25^ However, policies are not static; thus, we assigned policy categories based on the policy that was present at the time of article e-publication. For example, studies e-published by European Heart Journal (EHJ) in 2018 were categorized as ‘Required for Clinical trial’, whereas their studies e-published in 2023 were categorized as ‘Required’.

### Primary data analysis

A descriptive analysis was performed using R (version R 4.3.1) in RStudio (version 2023.09.1 + 494) to highlight DSS prevalence based on journal policy, study design, year of publication, funding source, article accessibility status, and study topic. Further, DSS prevalence was evaluated by journal and discipline. This was followed by a logistic regression analysis to estimate the odds of DSS inclusion in these disciplines. Next, we evaluated the trend of DSS presence by year for the four disciplines and cardiology journals.

We conducted a structured analysis to assess the influence of journal policies on DSS inclusion. For journals with policy changes during the study period (e.g. EHJ, JAMA Cardiology, JAMA Network, and The Lancet), articles were classified as ‘Pre-Policy Change’ or ‘Post-Policy Change’. European Heart Journal did not specify its policy change dates, but it followed ICMJE’s 2018 data sharing policies, classifying its policy then as ‘Required for Clinical Trials’. We inferred a policy change to ‘Required’ in 2020, when Oxford University Press announced a new data sharing policy on 29 September 2020.^26^ The Lancet was excluded from statistical analysis due to a 100% DSS inclusion rate before and after its policy implementation.

For journals that underwent policy changes, we evaluated policy effects on DSS prevalence by calculating DSS inclusion proportions within each policy period and used *χ*² or Fisher’s exact tests for significance testing. *Post hoc* analyses assessed whether DSS prevalence increased significantly following policy enforcement in specific journals.

A policy change sensitivity analysis was conducted to determine whether mandatory DSS policies independently influenced DSS inclusion while assessing other predictors like study design, funding source, article access, and impact factor. Due to convergence issues and unstable estimates in traditional logistic regression, we employed Firth’s penalized likelihood logistic regression to reduce small-sample bias and address data separation. We fitted three models:

1. Pre-Policy Model—Identified DSS inclusion predictors before policy enforcement.
2. Post-Policy Model—Identified DSS inclusion predictors after policy enforcement.
3. Policy Effect Model—Assessed whether policy enforcement was the primary driver of DSS inclusion, adjusting for confounders.

We also conducted logistic regression analyses to evaluate deviations from journal-mandated DSS policies and to assess factors influencing DSS presence in journals without a DSS policy. The deviation analysis excluded orthopaedic surgery (no ‘Required’ policies) and general medicine (lack of variability in DSS inclusion caused model instability), while the no-policy analysis excluded general medicine because all journals had a policy. Predictors included study design, funding source, article access, and discipline. Impact factor was excluded from both the deviation and no-policy models due to instability. Results were reported as odds ratios (ORs) with 95% confidence intervals (CIs).

### Qualitative analysis of data sharing statement themes

We performed a theme analysis of the DSS we extracted to quantify the variety of statements that were reported. We used ChatGPT-4o (https://openai.com/chatgpt/) to analyse all verbatim DSS in our pilot study sample. This resulted in 10 themes that characterize each DSS in the present study. Data sharing statement were deemed ineligible if data was unavailable without justification or when no instructions were given for acquiring data. This analysis was conducted for cardiology articles alone and for all disciplines.

### Verifying data accessibility

One DSS option is for researchers to provide data upon request. We were interested in assessing the frequency and ease with which authors who made this DSS type would share their information. To do this, we emailed the corresponding author of each article, asking two questions: (i) Are you able and willing to share the de-identified data necessary for replication of your primary outcome analysis? and (ii) If so, approximately when would you be prepared to send it for use in replication? The researchers were told that this request was part of a study evaluating the practicality and follow-through of DSS (see Supplementary data online for message template). Researchers were not asked to prepare or send their data. In the absence of a timely response, a follow-up request was sent 1 week later.

## Results

### Search results

Our search yielded 2426 returns across general medicine (1487) and cardiology (939). Additionally, we integrated 1959 returns from emergency medicine (875) and orthopaedic surgery (1084), resulting in 4384 records for potential analysis. After removing one duplicate, we excluded 1443 that did not meet study design inclusion criteria, leaving 2941 articles for full-text screening. Of these, 1937 (66%) did not include a DSS. Among the 1004 articles containing a DSS, 846 were eligible for the theme analysis (see Supplementary data online, *Figure S1*).

### General characteristics of included studies

The sample consisted of 2941 articles across multiple medical disciplines. The study designs varied, with clinical trials comprising the largest proportion, followed by cohort studies and other observational designs. Supplementary data online, *Table S1* summarizes the distribution of publication years, funding sources, and open access status across these disciplines. *Table 1* summarizes the distribution of publication years, funding sources, and open access status of the cardiology discipline.

**Table 1:** General characteristics of cardiology studies

Characteristic	Total = 910	DSS present	DSS absent
**Journal policy**			
Required	438 (48%)	396 (90%)	42 (10%)
No policy	310 (34%)	19 (6%)	291 (94%)
Required for clinical trials	162 (18%)	60 (37%)	102 (63%)
**Study design**			
Clinical trial	628 (69%)	367 (58%)	261 (42%)
Cohort (prospective or retrospective)	249 (27%)	92 (37%)	157 (63%)
Cross-sectional study	14 (2%)	7 (50%)	7 (50%)
Cost-effective analysis	8 (1%)	6 (75%)	2 (25%)
Case control study	6 (1%)	3 (50%)	3 (50%)
Case report/case series	5 (1%)	0 (0%)	5 (100%)
**Year**			
2020	283 (31%)	121 (43%)	162 (57%)
2021	223 (25%)	111 (50%)	112 (50%)
2022	189 (21%)	123 (65%)	66 (35%)
2023	142 (16%)	99 (70%)	43 (30%)
2019	73 (8%)	21 (29%)	52 (71%)
**Study topic**			
Treatment/interventional	675 (74%)	377 (56%)	298 (44%)
Screening/diagnostic	235 (26%)	98 (42%)	137 (58%)
**Funding source**			
Industry	310 (34%)	159 (51%)	151 (49%)
Multiple	244 (27%)	144 (59%)	100 (41%)
Government	193 (21%)	94 (49%)	99 (51%)
Private	84 (9%)	48 (57%)	36 (43%)
Not funded	59 (6%)	20 (34%)	39 (66%)
University	11 (1%)	5 (45%)	6 (55%)
Hospital	9 (1%)	5 (56%)	4 (44%)
**Article access**			
Open access	527 (58%)	212 (40%)	315 (60%)
Not open access	383 (42%)	263 (69%)	120 (31%)

### Data sharing statement inclusion across disciplines

**Figure 1:**
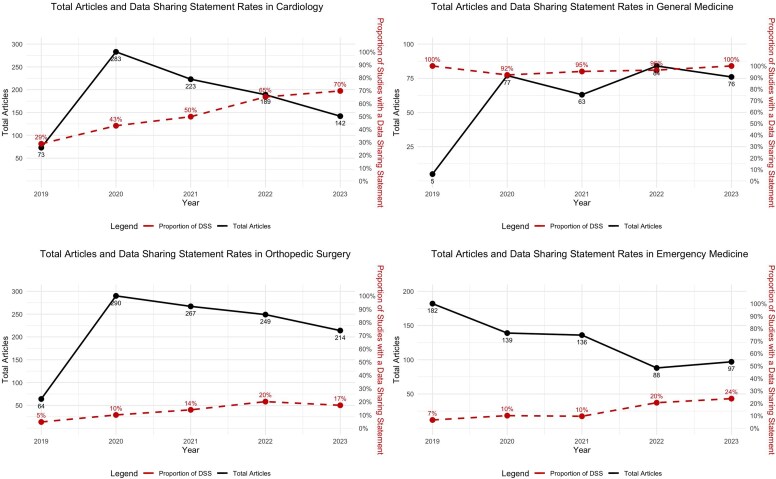
Data sharing statement trends across disciplines

In our DSS inclusion analysis across medical disciplines, we found substantial variability in reporting practices. General Medicine studies had the highest prevalence of DSS inclusion, with 96% (293/305) of manuscripts containing a DSS. In contrast, DSS were present in 52% (475/910) of Cardiology studies, 12% (80/642) of Emergency Medicine studies, and 14% (156/1084) of Orthopaedic Surgery studies. Trends over time revealed notable increases in DSS inclusion in Cardiology, rising from 29% in 2019 to 70% in 2023, while General Medicine maintained consistently high DSS rates (>90%). In contrast, DSS adoption in Emergency Medicine and Orthopaedic Surgery remained low, peaking at only 24% and 17% in 2023, respectively. Simple logistic regression analysis demonstrated that studies in General Medicine were significantly more likely to include a DSS compared with Cardiology (OR: 21.318, 95% CI: 11.773–38.604, *P* < .001). Conversely, DSS inclusion was significantly lower in Emergency Medicine (OR: 0.134, 95% CI: 0.102–0.178, *P* < .001) and Orthopaedic Surgery (OR: 0.155, 95% CI: 0.124–0.193, *P* < .001). These results are provided in further detail in *Figure 1* and *Table 2*.

**Table 2:** DSS inclusion across disciplines and journals

Discipline (*N*)	DSS present	DSS absent	Journal DSS policy
**Orthopaedic surgery (*N* *=* *1084*)** ^b^	**156 (14%)**	**928 (86%)**	
The Journal of Bone and Joint Surgery	83 (58%)	59 (42%)	No policy
Acta Orthopaedica	26 (37%)	44 (63%)	Recommended
Osteoarthritis and Cartilage	11 (23%)	37 (77%)	Recommended
The Bone and Joint Journal	20 (14%)	118 (86%)	No policy
The Spine Journal	7 (6%)	108 (94%)	Recommended
The American Journal of Sports Medicine	4 (4%)	103 (96%)	No policy
Clinical Orthopaedics and Related Research	2 (3%)	72 (97%)	No policy
The Journal of Arthroplasty	3 (2%)	184 (98%)	Recommended
Journal of Shoulder and Elbow Surgery	0 (0%)	122 (100%)	No policy
Arthroscopy	0 (0%)	81 (100%)	Recommended
**Cardiology (*N* = 910)**	**475 (52%)**	**435 (48%)**	
Circulation	278 (96%)	13 (4%)	Required
European Heart Journal	97 (58%)	69 (42%)	Required^a^
JAMA Cardiology	81 (57%)	62 (43%)	Required^a^
Journal of the American College of Cardiology	19 (6%)	291 (94%)	No policy
**Emergency medicine (*N* = 642)**	**80 (12%)**	**562 (88%)**	
World Journal of Emergency Surgery	28 (88%)	4 (12%)	Recommended
European Journal of Emergency Medicine	10 (14%)	60 (86%)	Required for clinical trials
Resuscitation	30 (10%)	271 (90%)	Recommended
Academic Emergency Medicine	5 (5%)	96 (95%)	No policy
Annals of Emergency Medicine	7 (5%)	131 (95%)	Required
**General medicine (*N* = 305)**	**293 (96%)**	**12 (4%)**	
The Lancet	67 (100%)	0 (0%)	Required^a^
New England Journal of Medicine	137 (98%)	3 (2%)	Required for clinical trials
JAMA Network	89 (91%)	9 (9%)	Required^a^

^a^Journals underwent a policy change during the window of time for which we selected our studies. For this case, all journals changed their policies from ‘Required for Clinical Trials’ to ‘Required.’.

^b^Bold rows indicate the group by which journals are classified.

### Impact of policy changes

**Figure 2:**
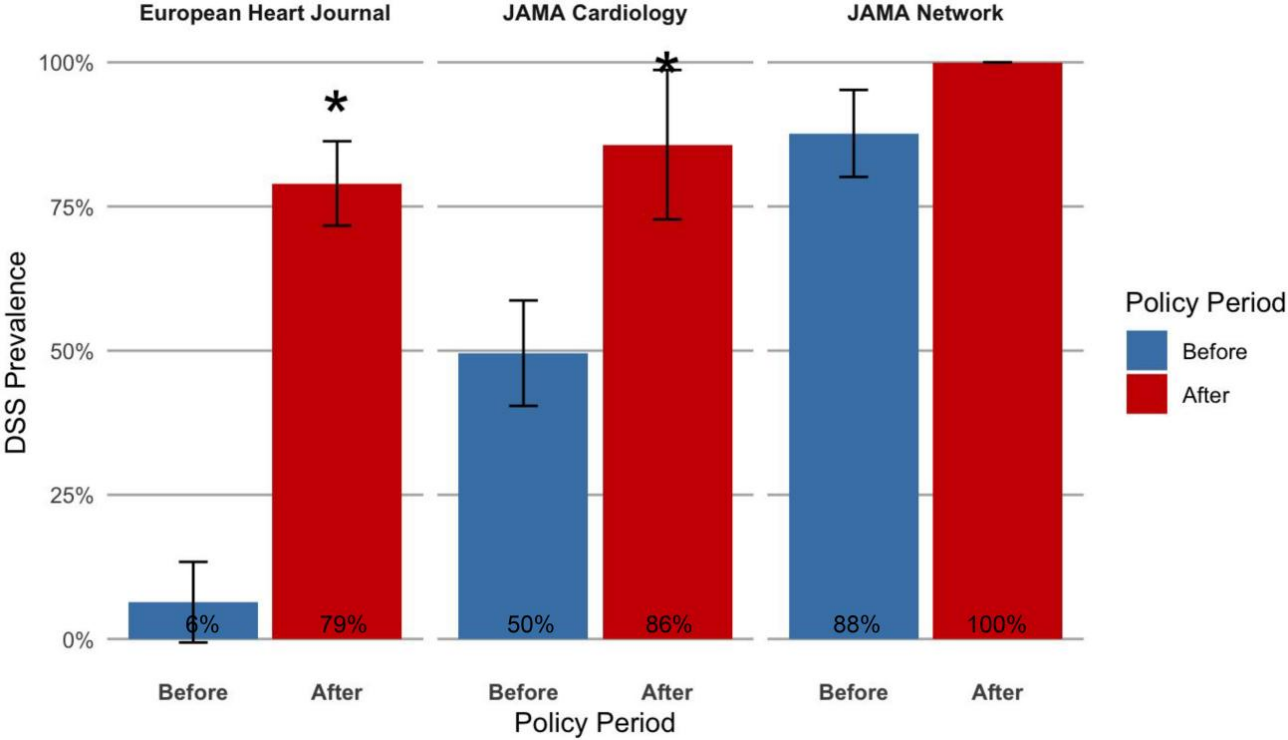
Comparison of data sharing statement prevalence before and after policy changes

Our unadjusted analysis demonstrated journal policy change had a significant effect on DSS inclusion. Pearson’s *χ*² test confirmed a strong association between policy status and DSS prevalence (*X*² = 248.740, df = 2, *P* < .001), indicating that the new mandatory DSS policies were associated with higher compliance rates. *Post hoc* analyses of JAMA Cardiology and EHJ found DSS inclusion significantly increased post-policy (OR 6.04, 95% CI 1.905–25.452, *P* < .001 for JAMA Cardiology; OR 53.465, 95% CI 15.258–289.038, *P* < .001 for EHJ). In contrast, JAMA Network showed no significant difference (OR = Inf, 95% CI 0.705–Inf, *P* = .106), as DSS prevalence was already high before the policy change. These results are illustrated in *Figure 2*. These results are illustrated by year in Supplementary data online, *Figure S2*.

### Policy change sensitivity analysis

Our policy change sensitivity analysis provided an adjusted model that confirmed policy enforcement drove DSS inclusion. Articles published before policy enforcement were 89% less likely to include a DSS than post-policy articles (OR 0.118, 95% CI 0.099–0.344, *P* < .001). Before policy enforcement, cohort studies were less likely than clinical trials to include a DSS (OR 0.012, 95% CI 0.001–0.052, *P* < .001), but this difference shrank post-policy (OR 2.152, 95% CI 0.499–13.408, *P* = 0.324). However, cohort studies remained less likely overall (OR 0.112, 95% CI 0.053–0.227, *P* < .001), suggesting barriers to DSS inclusion in cohort studies despite policy changes. Open-access status was consistently associated with greater DSS inclusion before (OR 0.286, 95% CI 0.135–0.590, *P* < .001) and after (OR 0.204, 95% CI 0.067–0.561, *P* = .002) policy enforcement. Non-funded studies were significantly less likely to include a DSS post-policy (OR 0.057, 95% CI 0.010–0.257, *P* < .001) and overall (OR 0.309, 95% CI 0.105–0.890, *P* = .030), suggesting financial support may aid compliance. Journal impact factor had no effect pre-policy but was linked to higher DSS inclusion post-policy (OR 1.032, 95% CI 1.011–1.059, *P* = .001) and overall (OR 1.011, 95% CI 1.000–1.023, *P* = .046), suggesting higher-impact journals may enforce policies more effectively. See *Table 3* for complete results.

**Table 3:** Policy change sensitivity analysis

Predictor	Pre-policy			Post-policy			Policy effect			Reference category
	OR	95% CI	*P*-value	OR	95% CI	*P*-value	OR	95% CI	*P*-value	
**Pre-policy enforcement**							**0.118**	**(0.099–0.344)**	**<.001** ^a^	Post-policy enforcement
**Study design**										
Cohort (prospective or retrospective)	**0.012**	**(0.001–0.052)**	**<.001**	2.152	(0.499–13.408)	.324	**0.112**	**(0.053–0.227)**	**<.001** ^a^	Clinical trial
**Funding source**										
Government	1.415	(0.601–3.429)	.428	0.924	(0.249–3.691)	.906	1.168	(0.592–2.331)	.655	Industry
Government, other	0.445	(0.171–1.137)	.091	0.897	(0.214–4.424)	.887	0.556	(0.264–1.177)	.124	Industry
Not funded	1.830	(0.324–13.572)	.502	**0**.**057**	**(0.010–0.257)**	**<**.**001**	**0**.**309**	**(0.105–0.890)**	.**030**	Industry
Private	1.824	(0.465–9.315)	.404	0.495	(0.116–2.396)	.361	1.141	(0.436–3.194)	.793	Industry
**Article access**										
Not open access	**0**.**286**	**(0.135–0.590)**	**<**.**001**	**0**.**204**	**(0.067–0.561)**	.**002**	**0**.**247**	**(0.138–0.434)**	**<**.**001**	Open access
**Impact factor**	1.001	(0.986–1.016)	.911	**1**.**032**	**(1.011–1.059)**	.**001**	**1**.**011**	**(1.000–1.023)**	.**046**	

^a^Bold rows indicate statistically significant findings (*P* < .050).

### Required policy deviations

Assessing adherence to mandatory DSS inclusion, government-funded (OR 0.307, 95% CI 0.098–0.961, *P* = .042), private-funded (OR 0.226, 95% CI 0.060–0.849, *P* = .028), and industry-funded studies (OR 0.249, 95% CI 0.080–0.780, *P* = .017) were more likely to comply with DSS mandates than unfunded studies. For disciplines, emergency medicine articles were significantly less likely to include a DSS than cardiology articles (OR 135.493, 95% CI 22.056–832.364, *P* < .001). However, open-access status did not significantly affect compliance (OR 0.524, 95% CI 0.265–1.034, *P* = .062). These findings provide insight into potential financial influence and discipline-specific trends regarding DSS requirements. This analysis is provided in Supplementary data online, *Table S2*.

### No policy journals data sharing statement

In journals without formal DSS policies, cohort studies were less likely to include a DSS than clinical trials (OR 0.282, 95% CI: 0.154–0.516, *P* < .001). Among funding sources, studies with industry and private support had significantly higher DSS inclusion rates than unfunded studies (OR 4.774, 95% CI 1.214–18.778, *P* = .025). However, open-access status was not significantly associated with DSS presence (OR 0.880, 95% CI 0.431–1.799, *P* = .727). Across medical disciplines, no significant differences in DSS inclusion were observed. Publisher policies that recommended DSS inclusion did not differ significantly in DSS adoption compared with publishers with no policy (OR 0.090, 95% CI 0.006–1.333, *P* = .080). These findings suggest that study design and funding sources may influence voluntary DSS inclusion without a formal journal policy. Supplementary data online, *Table S3* provides more detail regarding these findings.

### Theme analysis of data sharing statement in cardiology

The theme analysis presented in DSS in cardiology articles reveals several key trends. The *gatekeeper role* was the most common theme, present in 79% of DSS (347/441). Here, an individual controls the available data, granting researchers access upon request. Further, we classified over two-thirds (296/441; 67%) of DSS in cardiology journals as having *Conditional Data Availability*, meaning the data would be accessible only under specific conditions. More studies placed their data under structured access platforms (58/441; 13%) rather than open-access repositories (43/441; 10%), and even fewer made data immediately accessible within their manuscript or supplementary files (10/441; 2%). Conditional timing, contingent on publication dates or the achievement of specific milestones, constituted 19% of DSS in our sample (85/441). Ethical and legal constraints played a role in 15% of DSS (66/441), while corporate ownership was a factor in 5% (20/441). Additional themes can be found in *Table 4*. Combined theme analysis of all disciplines can be found in Supplementary data online, *Table S4*.

**Table 4:** Data sharing statement themes (*N* = 441, multiple themes may be present)

Theme	Definition	Frequency of themes observed	Frequency of theme combination	Implications for practice
Gatekeeper role	Access controlled by individual or group	347 (79%)	613	Allows for oversight, ethical review, and accountability.
Conditional data availability	Data is shared only if certain conditions are met.	296 (67%)	565	Enables ethical sharing while limiting risk or misuse.
Privacy concerns	Data sharing is limited to protect participant privacy.	115 (26%)	333	Supports compliance with privacy laws and ethical standards.
Conditional timing for access and specified availability date	Access is delayed until a specific date or milestone.	85 (19%)	243	Helps manage resources and protect ongoing analyses.
Ethical and legal constraints	Legal or ethical rules prevent sharing.	66 (15%)	167	Ensures adherence to policy and protects participants or institutions.
Structured access platform and access mechanism	Access is managed via a formal platform or repository.	58 (13%)	222	Facilitates secure sharing and research reproducibility.
Open data sharing and public repository use	Data is deposited in a public repository.	43 (10%)	136	Promotes transparency and reuse of data.
Transparency and accessibility challenges	Authors acknowledge competing needs for openness and protection.	27 (6%)	131	Highlights trade-offs and limits to data access.
Corporate ownership	Data is owned by a sponsor or company.	20 (5%)	37	May limit sharing to protect intellectual property or legal interests.
Immediate data accessibility	Data is included directly in the article or supplements.	10 (2%)	1	Allows full transparency and immediate reuse by others.

### Responses to data requests

To evaluate DSS feasibility and execution, we contacted corresponding authors who had stated their data could be accessed upon request. We emailed 419 authors, and 25 automated responses were received indicating temporary unavailability, while an additional 14 were undeliverable to the corresponding authors. Only 137 authors responded to our emails (33%) and were categorized as follows: 43 (31%) were able or willing to share data for replication of primary outcome analysis, 49 (36%) committed to sharing their data after specific conditions were met, 26 (19%) refused to share data, and 6 (4%) opted out of the study. For each contact that reported conditional availability, 26 (53%) cited ethical or legal restrictions concerning their ability to share data, and 15 (31%) cited that data was proprietary or intellectual property. The responses refusing to share data reported ethical or legal restriction (10/26; 38%), intellectual property (4/26; 15%), data use ongoing (3/26; 12%), confidentiality concerns (3/26; 12%), resource or time constraints (2/26; 8%), or did not specify (4/26; 15%) (see Supplementary data online, *Table S5*). All author responses can be found in supplementary materials.

## Discussion

DSS inclusion varies widely across journals and disciplines, reflecting progress and ongoing challenges in achieving data sharing. Some researchers and policymakers advocate for increased data sharing, citing benefits such as improved reproducibility, enhanced collaboration, and greater trust in scientific findings. Others, however, emphasize complexities and barriers that hinder widespread data sharing, including privacy concerns, data ownership conflicts, and the logistical burdens associated with maintaining and distributing datasets. This discussion situates our findings within this broader debate, acknowledging the competing perspectives that shape data sharing practices in cardiology research.

Among the cardiology journals analysed, the prevalence of DSS varied substantially, with journal policies playing a key role. Circulation consistently reported DSS in over 90% of its published articles, while EHJ and JAMA Cardiology showed increasing DSS inclusion rates over time, reaching 96% and 90%, respectively, in 2023. In contrast, the Journal of the American College of Cardiology, which lacks a mandatory DSS policy, had significantly lower DSS inclusion, with only 6% of its articles containing a DSS. These differences align with journal policies, as our policy change sensitivity analysis revealed that DSS inclusion increased by almost 90%. These findings suggest that policy enforcement, rather than discipline-specific norms alone, is a major driver of DSS adoption. While requiring DSS fosters transparency and promotes open research, challenges remain, including ethical and logistical barriers that researchers face in making data publicly available.^8,14^

Funding sources also appeared to be associated with DSS inclusion in our study. Articles supported by industry, private organizations, or multiple funding sources were more likely to include DSS than unfunded studies. This trend may reflect expectations from funding bodies to promote transparency or provide resources that facilitate data sharing practices.^9,27^ While advocates urge funding agencies to mandate DSS for accountability, critics cite conflicts with commercial interests, as industry-sponsored research can involve complexity with proprietary data and intellectual property rights.^28,29^ Another significant consideration in industry-sponsored trials is the extent to which principal investigators (PIs) and steering committee members can access the full dataset. The industry sponsor often retains control over the trial database, providing PIs with only pre-specified primary and selected secondary analyses rather than full access to raw data.^30,31^ As a result, most co-authors rely on the first author to confirm data integrity on behalf of the entire research team, raising concerns about independent researchers’ ability to verify results, conduct exploratory analyses, or assess the full breadth of trial data. While industry stakeholders argue that data protection measures are necessary to safeguard proprietary information and maintain regulatory compliance, critics contend that limited access undermines secondary data use.^10,32^ Addressing these concerns requires a balance between protecting commercially sensitive data and ensuring that trial investigators have adequate access to perform independent evaluations.^13,33^ Increasingly, regulatory agencies and journals have called for more explicit policies regarding industry-PIs data access, but variability in implementation persists.^34^

The introduction of the ICMJE's 2018 policy requiring DSS for clinical trials was a significant step towards data sharing. This policy reinforces the ethical argument that clinical trial participants assume risks under the expectation that their data will contribute to broader scientific knowledge.^35^ However, this mandate does not extend to observational and cohort studies, which exhibit lower DSS inclusion rates in our analysis. While some argue that all study designs should be subject to the same transparency standards, others caution that observational data may involve complex ethical and logistical considerations that complicate sharing.^14,29^ Concerns regarding patient confidentiality, institutional data policies, and potential misuse of data further contribute to the reluctance to extend DSS mandates universally.^15,28,36^ Data sharing across international borders presents additional challenges, with differences between Europe and the United States serving as an example. Regulatory frameworks such as Europe’s General Data Protection Regulation (GDPR) impose strict requirements on cross-border data transfers, whereas the US follows a sector-specific approach with regulations like HIPAA governing medical data.^37^ These differences create legal and logistical hurdles, as data sharing agreements must align with multiple, sometimes conflicting, policies. Additionally, variability in patient consent requirements complicates collaboration, as European regulations often require more explicit, narrowly defined consent than U.S. research protocols typically allow.^37^ Further, ethical concerns, especially among European institutions, stem from GDPR restrictions on consent, purpose limitation, and data minimization in the secondary use of patient data.^37^ Beyond regulatory barriers, technical incompatibilities—including differences in electronic health record systems, data coding standards, and repository structures—hinder seamless data integration. Addressing these barriers requires harmonized legal agreements, standardized data formats, and governance frameworks that balance privacy protections with scientific collaboration^34^ (*Structured Graphical Abstract*).

Despite the increasing DSS prevalence in cardiology research, a significant gap remains between stated commitments and actual data availability. Many authors who indicate that data is ‘available upon request’ are difficult to engage,^18^ and corresponding authors—who often control dataset access—may unintentionally hinder transparency due to the demands of their position.^27,31^ These individuals must navigate institutional policies, ensure proper de-identification, address ethical concerns, and coordinate with co-authors or funding bodies,^34^ creating logistical challenges that can delay or prevent data sharing. In some cases, data simply cannot or should not be shared due to ethical constraints, proprietary restrictions, or regulatory requirements. Additionally, some authors feel pressured to declare data availability to avoid bias in the publication process, fearing that openly declining to share data could jeopardise manuscript acceptance. As a result, they may claim compliance with data sharing policies despite lacking the resources or capacity to follow-through.^29,32^ Moreover, concerns have been raised about the potential misuse of shared datasets by individuals lacking expertise in the study domain, which could lead to misinterpretation of findings.^36,38^ Effective data sharing policies should not only promote best practices but also allow authors to disclose legitimate barriers, such as ethical constraints, inadequate infrastructure, or resource limitations.^14^ Simultaneously, fostering collaboration between original authors and secondary analysts—supported by proper documentation and ethical oversight—can help mitigate the risks of data misuse while advancing responsible and transparent research.^33^

Stakeholders propose various strategies to balance data sharing goals with practical challenges. Some advocate for stricter journal policies, such as requiring persistent dataset identifiers (e.g. DOIs) and conducting random audits to enforce compliance.^29,30^ These measures, they argue, would deter non-compliance and enhance scientific rigour. Others caution that such requirements may overburden researchers, particularly those handling sensitive data or operating with limited resources.^13^ Punitive measures like retractions or public notices could encourage adherence and deter submissions to journals with strict DSS policies. Despite these challenges, structured data sharing initiatives have demonstrated significant scientific value. The NIH-funded SPRINT and TOPCAT trials exemplify how publicly available datasets can drive secondary analyses that expand scientific knowledge. Reanalysis of SPRINT data led to new insights into blood pressure management, while investigations using TOPCAT data refined heart failure treatment strategies across patient sub-groups.^39,40^ These cases highlight how structured data sharing policies foster collaboration, generate new discoveries, and improve patient care. Expanding and incentivizing such models across cardiology research could enhance transparency and scientific impact.^10^

Beyond policy enforcement, cultural attitudes towards data sharing significantly shape researcher behaviour. Positive reinforcement strategies—such as academic recognition, citation incentives, and co-authorship opportunities—may encourage greater participation.^36^ The FAIR (Findable, Accessible, Interoperable, and Reusable)^34^ and TOP (Transparency and Openness Promotion) guidelines offer frameworks to standardise data sharing expectations.^41^ Encouraging collaboration rather than relying solely on compliance measures can improve adoption rates, particularly in multi-institutional and cross-disciplinary research, where shared datasets reduce redundancy and foster knowledge exchange.^8,19,33^

Recognising data contributions through formal collaborations and funding opportunities has been suggested to complement policy-driven incentives.^15,28^ Given the time, effort, and potential legal costs associated with data sharing, some suggest that multiple stakeholders—including funding bodies, research institutions, and journals—could play a role in supporting these activities. Some argue that journals should facilitate data sharing through clearer guidance, infrastructure, or standardised agreements, while others contend that financial and administrative support should come primarily from research funders or data-generating institutions.^12,30^ Collaborative funding models, in which secondary analysts apply for grants alongside original investigators, have been proposed to distribute costs and encourage cooperation.^29^ Efforts to address financial barriers may benefit from ongoing discussions among funders, institutions, and publishers to explore sustainable models that balance transparency with feasibility.^34^ Ultimately, fostering a culture of responsible data sharing—through supportive policies, researcher incentives, and sustainable funding models—may be key to bridging the gap between DSS commitments and real-world data availability.

### Strengths and limitations

Our study’s strengths include, in accordance with the Cochrane Handbook for systematic reviews, a thorough, independent data extraction.^42^ All data, protocols, analysis scripts, and extraction forms were uploaded to OSF to promote transparency. Further, our cross-disciplinary approach, comparing cardiology journals with those in general medicine, emergency medicine, and orthopaedic surgery, provides broader context for DSS inclusion rates. Additionally, access to raw data from these previous studies ensured methodological consistency and adherence to PRISMA guidelines enhanced reproducibility. However, limitations exist. Using Journal Citation Reports^TM^ to select top journals may introduce selection bias, and PubMed filters could have excluded relevant articles. Next, excluding studies without novel primary data or those outside the timeframe limits generalizability. Our comparative approach, while valuable, introduces potential biases. Journal representation varied across fields, with cardiology and general medicine comprising fewer journals with consistent DSS policies, potentially inflating perceived DSS inclusion compared with the more diverse journals in emergency medicine and orthopaedic surgery. Qualitative analysis of DSS themes is contingent upon the quality and detail of statements provided by authors, which can vary. A key limitation of our study is its reliance on DSS within published articles, which may skew findings towards successful data sharing efforts. Many data sharing attempts fail due to institutional policies, proprietary concerns, or unwillingness from data custodians. Yet, these challenges are rarely documented in publications, leading to failed efforts being under-represented. Even when data sharing is formally stated, practical barriers such as intellectual property constraints and privacy risks often impede actual data exchange. Additionally, while our team has experience with data sharing in meta-research, we recognize that clinical research presents unique hurdles—including patient privacy regulations and institutional policies—that extend beyond our direct experience. Despite these limitations, our study provides a systematic assessment of data sharing practices in leading cardiology journals, offering insight into broader trends and challenges.

## Conclusions

Our study demonstrates that journal policies play a key role in increasing DSS inclusion in research. However, barriers such as ethical concerns, data ownership, and logistical challenges persist. Funding sources also influence DSS presence, with industry and privately funded studies more likely to include DSS than unfunded research. Despite DSS inclusion, a disconnect remains between stated data availability and the actual ability to access shared data, with many requests going unanswered or unfulfilled. Ensuring sustainable and equitable data sharing efforts requires a collaborative approach, where funding agencies, institutions, and journals provide the necessary resources and infrastructure to support researchers. Neither individual investigators nor journals alone should bear the financial and logistical burdens of data sharing. Instead, those requesting access to data or seeking to share it should take primary responsibility for securing the financial and administrative resources needed to facilitate responsible data access. Data sharing initiatives should be designed with practical constraints in mind, offering financial support, clear guidance, and incentives such as citation benefits and structured repositories. Future efforts should focus on balancing transparency with feasibility, ensuring that policy enforcement is aligned with the resources available to all stakeholders involved in the data sharing process.

## Supplementary Material

ehaf359_Supplementary_Data

## Declarations

### Data Availability

In line with promoting open science practices, reproducibility, and transparency, we have made our protocol (including all its versions), analysis scripts, research data, and other study materials available on the Open Science Framework (OSF). https://osf.io/9xgqr/?view_only=845c21765d844ae0912d89fb86d2075e.
