# Supplementary material for: Data sharing statements: impact of journal policies across clinical research disciplines
Source: Eur Heart J. 2025 May 30;47(27):3610–21. doi: 10.1093/eurheartj/ehaf359 (PMC13364081; doi:10.1093/eurheartj/ehaf359)

**Supplement Table 1: General Characteristics of All Studies**

| **Characteristic** | **N (%)**  **Total = 2941** | **DSS**  **Present**  **N (%)** | **DSS**  **Absent**  **N (%)** |
| --- | --- | --- | --- |
| **Journal Policy** |  |  |  |
| *No Policy* | 994 (34%) | 133 (13%) | 861 (87%) |
| *Recommended* | 834 (28%) | 105 (13%) | 729 (87%) |
| *Required* | 657 (22%) | 484 (74%) | 173 (26%) |
| *Required for Clinical Trials* | 456 (16%) | 282 (62%) | 174 (38%) |
| **Study Design** |  |  |  |
| *Clinical Trial* | 1,856 (63%) | 800 (43%) | 1,056 (57%) |
| *Cohort (Prospective or Retrospective)* | 971 (33%) | 180 (19%) | 791 (81%) |
| *Cross-Sectional Study* | 57 (2%) | 9 (16%) | 48 (84%) |
| *Cost-effective analysis* | 22 (1%) | 10 (45%) | 12 (55%) |
| *Case Control Study* | 19 (1%) | 5 (26%) | 14 (74%) |
| *Case Report/Case Series* | 14 (0%) | 0 (0%) | 14 (100%) |
| *Qualitative Study* | 2 (0%) | 0 (0%) | 2 (100%) |
| **Year** |  |  |  |
| *2020* | 790 (27%) | 236 (30%) | 554 (70%) |
| *2021* | 690 (23%) | 222 (32%) | 468 (68%) |
| *2022* | 609 (21%) | 271 (44%) | 338 (56%) |
| *2023* | 528 (18%) | 234 (44%) | 294 (56%) |
| *2019* | 324 (11%) | 41 (13%) | 283 (87%) |
| **Study Topic** |  |  |  |
| *Treatment/Interventional* | 2,201 (75%) | 838 (38%) | 1,363 (62%) |
| *Screening/Diagnostic* | 740 (25%) | 166 (22%) | 574 (78%) |
| **Funding Source** |  |  |  |
| *Multiple* | 593 (20%) | 249 (42%) | 344 (58%) |
| *Government* | 565 (19%) | 221 (39%) | 344 (61%) |
| *Industry* | 562 (19%) | 299 (53%) | 263 (47%) |
| *Not Listed* | 539 (18%) | 39 (7%) | 500 (93%) |
| *Not funded* | 312 (11%) | 74 (24%) | 238 (76%) |
| *Private* | 249 (8%) | 96 (39%) | 153 (61%) |
| *University* | 61 (2%) | 13 (21%) | 48 (79%) |
| *Hospital* | 60 (2%) | 13 (22%) | 47 (78%) |
| **Article Access** |  |  |  |
| *Not Open Access* | 1,520 (52%) | 449 (30%) | 1,071 (70%) |
| *Open Access* | 1,421 (48%) | 555 (39%) | 866 (61%) |

**Supplement Table 2: Factors Associated with DSS Absence in Journals with a “Required” DSS Policy**

| **Entity** | **Odds Ratio** | **OR CI Lower** | **OR CI Upper** | **P value** | **Reference** |
| --- | --- | --- | --- | --- | --- |
| **Study Design** |  |  |  |  |  |
| *Cohort (Prospective or Retrospective)* | 1.092 | 0.502 | 2.375 | 0.824 | Clinical Trial |
| *Cross-Sectional Study* | 2.210 | 0.242 | 20.206 | 0.482 | Clinical Trial |
| **Funding Source** |  |  |  |  |  |
| *Government* | **0.307** | **0.098** | **0.961** | **0.042** | Not Funded |
| *Government, Other* | 0.303 | 0.091 | 1.011 | 0.052 | Not Funded |
| *Hospital* | 0.213 | 0.015 | 2.961 | 0.250 | Not Funded |
| *Industry* | **0.249** | **0.080** | **0.780** | **0.017** | Not Funded |
| *Industry, Private* | 0.208 | 0.038 | 1.142 | 0.071 | Not Funded |
| *Private* | **0.226** | **0.060** | **0.849** | **0.028** | Not Funded |
| *University* | 0.367 | 0.034 | 3.973 | 0.410 | Not Funded |
| **Article Access** |  |  |  |  |  |
| *Open Access* | 0.524 | 0.265 | 1.034 | 0.062 | Not Open-Access |
| **Discipline** |  |  |  |  |  |
| *Emergency Medicine* | **135.493** | **22.056** | **832.364** | **<0.001** | Cardiology |

1. Only articles that were published in journals by journals with a “Required” policy were incorporated into this analysis. General Medicine articles were excluded due to little variation in their DSS inclusion, which caused instability to the model. No Orthopedic Surgery journals had “Required” policy for DSS inclusion, explaining its absence from the table.

**Supplement Table 3: Factors Associated with DSS Presence in Journals with “No Policy”**

| Entity | Odds Ratio | OR CI Lower | OR CI Upper | P value | Reference |
| --- | --- | --- | --- | --- | --- |
| **Study Design** |  |  |  |  |  |
| *Cohort (Prospective or Retrospective)* | **0.282** | **0.154** | **0.516** | **<0.001** | Clinical Trial |
| *Cross-Sectional Study* | 0.500 | 0.046 | 5.378 | 0.567 | Clinical Trial |
| **Funding Source** |  |  |  |  |  |
| *Government* | 0.720 | 0.280 | 1.850 | 0.495 | Not Funded |
| *Government, Other* | 0.952 | 0.357 | 2.536 | 0.921 | Not Funded |
| *Hospital* | 3.082 | 0.761 | 12.477 | 0.115 | Not Funded |
| *Industry* | 1.584 | 0.621 | 4.041 | 0.336 | Not Funded |
| *Industry, Private* | **4.774** | **1.214** | **18.778** | **0.025** | Not Funded |
| *Not Listed* | 0.678 | 0.284 | 1.620 | 0.382 | Not Funded |
| *Private* | 0.533 | 0.171 | 1.663 | 0.279 | Not Funded |
| *University* | 1.024 | 0.116 | 9.029 | 0.983 | Not Funded |
| **Article Access** |  |  |  |  |  |
| *Open Access* | 0.880 | 0.431 | 1.799 | 0.727 | Not Open-Access |
| **Discipline** |  |  |  |  |  |
| *Emergency Medicine* | 1.186 | 0.021 | 67.510 | 0.934 | Cardiology |
| *Orthopedic Surgery* | 0.496 | 0.017 | 14.210 | 0.682 | Cardiology |
| **Publisher Policy** |  |  |  |  |  |
| *Recommended* | 0.090 | 0.006 | 1.333 | 0.080 | No Policy |

**Supplement Table 4. Data Sharing Statement Themes for All Disciplines (N = 850, multiple themes may be present)**

| **Theme** | **Definition** | **Frequency of Themes Observed** | **Frequency of Theme Combination** | **Implications for Practice** |
| --- | --- | --- | --- | --- |
| *Gatekeeper Role* | Access controlled by individual or group | 671  (79%) | 1385 | Allows for oversight, ethical review, and accountability. |
| *Conditional Data Availability* | Data is shared only if certain conditions are met. | 592  (70%) | 1315 | Enables ethical sharing while limiting risk or misuse. |
| *Privacy Concerns* | Data sharing is limited to protect participant privacy. | 321  (38%) | 946 | Supports compliance with privacy laws and ethical standards. |
| *Conditional Timing for Access and Specified Availability Date* | Access is delayed until a specific date or milestone. | 264  (31%) | 781 | Help manage resources and protect ongoing analyses. |
| *Ethical and Legal Constraints* | Legal or ethical rules prevent sharing. | 141  (17%) | 395 | Ensures adherence to policy and protects participants or institutions. |
| *Structured Access Platform and Access Mechanism* | Access is managed via a formal platform or repository. | 121  (14%) | 444 | Facilitates secure sharing and research reproducibility. |
| *Corporate Ownership* | Data is owned by a sponsor or company. | 92  (11%) | 237 | May limit sharing to protect intellectual property or legal interests. |
| *Open Data Sharing and Public Repository Use* | Data is deposited in a public repository. | 65  (8%) | 195 | Promotes transparency and reuse of data. |
| *Transparency and Accessibility Challenges* | Authors acknowledge competing needs for openness and protection. | 35  (4%) | 164 | Highlights trade-offs and limits to data access. |
| *Immediate Data Accessibility* | Data is included directly in the article or supplements. | 20  (2%) | 12 | Allows full transparency and immediate reuse by others. |

^a^NHLBI = National Heart, Lung, and Blood Institute

Supplement Figure 1: Prisma Flow Diagram


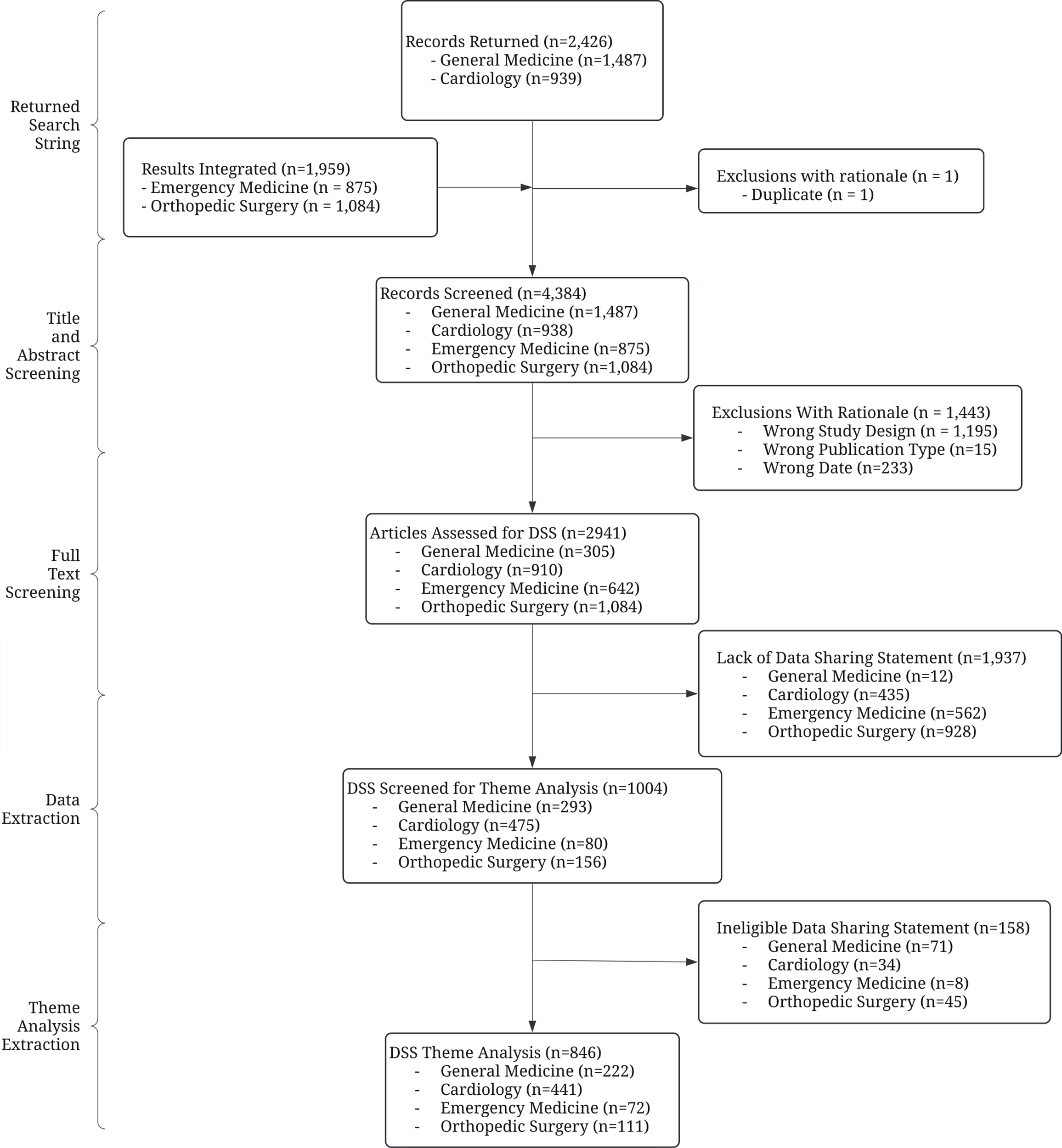


Supplement Figure 2: Data Sharing Statement Rates by Journal


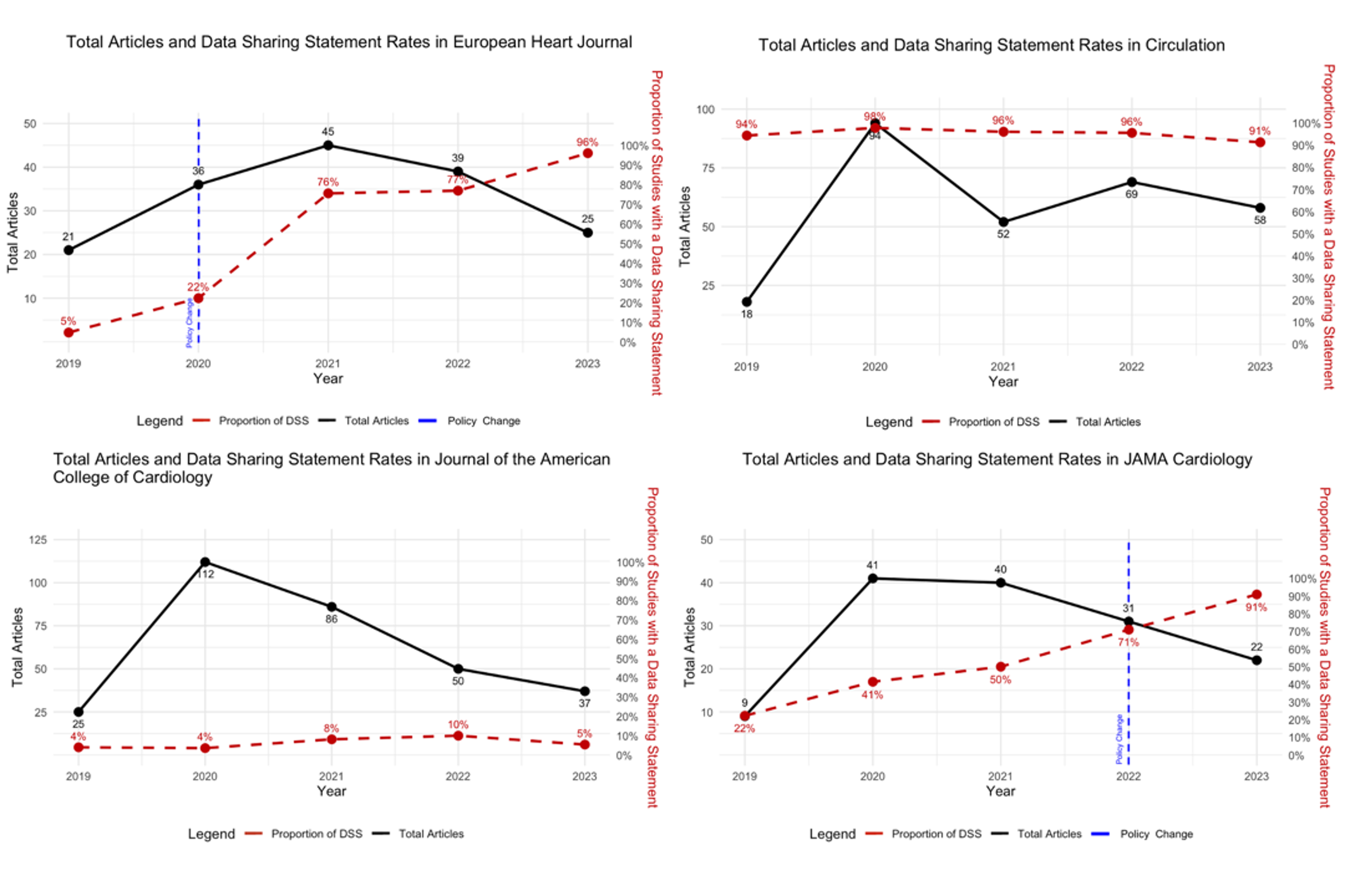

Supplement: ehaf359_Supplementary_Data [file ehaf359_supplementary_data.zip › RR4 Supp_T1-4_F1-2.docx]
