# Supplementary material for: Data sharing statements: impact of journal policies across clinical research disciplines
Source: Eur Heart J. 2025 May 30;47(27):3610–21. doi: 10.1093/eurheartj/ehaf359 (PMC13364081; doi:10.1093/eurheartj/ehaf359)
Supplement: ehaf359_Supplementary_Data [file ehaf359_supplementary_data.zip › Supplementary_data_online_Table_S5-Email_Responses.docx]

**Supplementary Table 5. Corresponding Author Response to Data Request**

| **Response** |  |
| --- | --- |
| Did not respond to request | 282/419 (67%) |
| Responded to request | 137/419 (33%) |
| **Did not respond to request (n=282)** |  |
| Undeliverable | 14/282 (5%) |
| No response | 243/282 (86%) |
| Out of office | 25/282 (9%) |
| **Responded to request (n=137)** |  |
| Willing to share data | 43/137 (31%) |
| Would share data with condition | 49/137 (36%) |
| Refused to share data | 26/137 (19%) |
| Acknowledged, not answered | 13/137 (9%) |
| Opted Out of Study | 6/137 (4%) |
| **Reason for conditional status of data (n=49)** |  |
| Ethical and legal restrictions | 26/49 (53%) |
| Intellectual Property and Proprietary Data | 15/49 (31%) |
| Confidentiality and privacy concerns | 2/49 (4%) |
| Concerns about misuse or misinterpretation | 2/49 (4%) |
| Incomplete or Unready Data | 2/49 (4%) |
| Moved to structured access repository | 1/49 (2%) |
| Resource and Time Constraints | 1/49 (2%) |
| **Reason for refusal to share data (n=16)** |  |
| Ethical and legal restrictions | 10/26 (38%) |
| Intellectual property and proprietary data | 4/26 (15%) |
| Did not specify | 4/26 (15%) |
| Confidentiality and Privacy Concerns | 3/26 (12%) |
| Data sensitivity and impact on ongoing research | 3/26 (12%) |
| Resource and time constraints | 2/26 (8%) |
| **Time to fulfill data request (n=90)** |  |
| Unspecified | 46/90 (51%) |
| Immediately accessible | 13/90 (14%) |
| Less than one month | 10/90 (11%) |
| Greater than one month | 4/90 (4%) |
| Greater than two months | 5/90 (6%) |
| Greater than three months | 12/90 (13%) |
